# Actinomycosis

**Published:** 1897-06

**Authors:** B. F. Kaupp

**Affiliations:** Kansas City, Mo.


### ACTINOMYCOSIS.

By B. F. Kaupp, D.V.S.,
KANSAS CITY, MO.

Actinomycosis is an affection characterized by the presence in the animal tissues of a vegetable parasite, or fungus, known as actinomyces, which causes a chronic inflammatory process usually resulting in abscess. This disease has been called by other terms, such as "lumpy jaw," "big jaw," and "swelled head." The disease, which was first found in cattle, was described by Bollinger in 1877, and in 1878 was also found in man. It was not thoroughly understood until about 1882. Clumps of the organism producing this disease can be seen in the pus by the unaided eye; they are small bodies, varying in size from a pin-point to a millet-seed, and in color are yellowish-white. Under the microscope they appear as clusters of straight or wavy branching threads, and also as radiating prolongations, thick and club-shaped, whose club-ends are directed out toward the circumference, and which radiate from the centre and give them somewhat the appearance of a sunflower. Cultures of the ray-fungus are made with difficulty; the medium upon which they are grown is agar-agar, egg-albumin, blood-serum, and gelatin.

It is claimed that attempts to transfer the actinomyces from one animal to another by mixing it in the food have not been successful, but that inoculations have been successfully made by introducing the granules into the peritoneal cavity of rabbits, and by injecting the infective material beneath the skin into the veins and into the abdominal cavity of calves.

Dr. Warren, of Boston, in his *Surgical Pathology*, gives us one point worth noting, and that is that "this disease has never been found in carnivorous animals." The most frequent route of infection is through the mouth, entering cavities of decayed teeth or abraded surfaces in the mouth or other portions of the digestive tract. It may also be conveyed through abraded surfaces on the skin. The above author also states that "after once having gained a foothold, the parasite is said to be conveyed to distant parts of the body through the blood-currents, and not through the lymphatics."

The actinomycotic abscesses when cut open appear to be lined with a granular membrane, and usually contain a thick, creamy pus, in which the yellowish granular bodies float. The tumors are surrounded by thick capsules, the surface of which is fibrous. When the actinomyces begin to develop in the tissue a growth of sarcomatous connective tissue is built up around them. Nature, probably, in this way tends to retard the growth. The cells nearest the centre of the growth undergo fatty degeneration, and in this way form small abscesses; the intervening tissue breaking down, they communicate with each other, forming larger ones.

We find actinomycotic tumors affecting the superior maxillary bone; the maxillary sinuses may be filled partially or completely with actinomycotic growth, which may possibly extend into the frontal sinuses. These sinuses may also contain pus. We may find large growths upon one or both branches of the inferior maxillary bone. This disease may affect one or both of the posterior pharyngeal lymphatic glands, the affected glands sometimes reaching the size of a goose-egg, and containing in the centre a thick and more or less granular fluid. The submaxillary and other lymphatic glands of the head are sometimes affected. The parotid gland is sometimes the part invaded, probably by the germ finding its way to that gland through Steno's duct. We find this disease affecting the soft structures of the head and occasionally the tongue. The lungs are sometimes the seat of the disease, where we find in one or more lobes masses of actinomycotic growth.

On the European continent this disease appears to most frequently affect the tongue, while in this country it is most often found affecting the glands and bones of the head. When the bony tissues are invaded by this vegetable germ we notice the characteristic proliferation of the cells of the osseous and periosseous tissues, which often attain great dimensions. By reason of the activity of the fungus, the bone and its coverings are supplanted by a new growth of fibrous tissue, inclosing masses of granular tissue in which are found the granular bodies or fungi. Externally these abscesses or tumors appear to be irregularly oval in shape, and we may possibly see several of them on the superior maxillary and other bones of the head.

If not arrested in its growth, the actinomyces erode one or more channels to the cutaneous and mucous surfaces, through which the fungus and the fluid in which it floats escape, falling into the mouth-cavity or on to the ground if in pasture; on the woodwork, etc., if the animal be kept in a stable. When mucous and cutaneous surfaces are invaded by the actinomyces it is manifested by fungous granulations, which have been described as ulcerating granuloma. These masses emit an offensive necrotic odor, and have a repulsive appearance.

On the European continent this disease is known as "wooden tongue." It is quite common, and is said to be permanently cured by the iodide of potassium treatment. This treatment was recommended by a German veterinarian about the year 1880. It appears that no one successfully treated actinomycosis of the jaw in this country until 1892, when Dr. Norgaard, veterinary inspector in the Bureau of Animal Industry, first tried the experiment with success. Since then many practitioners have reported cures.

During the past ten months I have had considerable experience in holding post-mortems upon actinomycotic cattle at one of the largest abattoirs of this city. The total number of post-mortems during this time aggregated about a thousand. Of these about 30 per cent. had the left submaxilla affected, and 20 per cent. the right; 20 per cent. the right superior, and 20 per cent. the left superior maxillary bones; 5 per cent. were in the soft structures only; 5 per cent. in both the right and left superior maxillary bones; and about 1 per cent. in the parotid glands. In about 4 per cent. actinomycotic lesions were found in the lungs, and about 5 per cent. in the posterior pharyngeal lymphatic glands; and in about 40 per cent. the submaxillary lymphatic glands were more or less involved. The mesenteric lymphatic glands and the liver are sometimes affected. In about 20 per cent. of the total number the disease was extensive or in advanced stages.

